# A Case-Control Study of Salivary Redox Homeostasis in Hypertensive Children. Can Salivary Uric Acid be a Marker of Hypertension?

**DOI:** 10.3390/jcm9030837

**Published:** 2020-03-19

**Authors:** Mateusz Maciejczyk, Katarzyna Taranta-Janusz, Anna Wasilewska, Agnieszka Kossakowska, Anna Zalewska

**Affiliations:** 1 Department of Hygiene, Epidemiology and Ergonomics, Medical University of Bialystok, 2c Mickiewicza Street, 15-233 Bialystok, Poland; 2 Department of Pediatrics and Nephrology, Medical University of Bialystok, 24a M. Sklodowskiej-Curie Street, 15-274 Bialystok, Poland; katarzyna.taranta@wp.pl (K.T.-J.); annwasil@interia.pl (A.W.); 3 Experimental Dentistry Laboratory, Medical University of Bialystok, 24a M. Sklodowskiej-Curie Street, 15-274 Bialystok, Poland; a_kossak@op.pl (A.K.); azalewska426@gmail.com (A.Z.)

**Keywords:** hypertension, salivary biomarkers, oxidative stress, redox homeostasis, salivary antioxidants

## Abstract

Oxidative stress plays a critical role in the pathogenesis of hypertension; however, there are no data on salivary redox homeostasis and salivary gland function in children with hypertension. A total of 53 children with hypertension and age- and sex-matched controls were classified for the study. The antioxidant barrier and oxidative/nitrosative stress were evaluated in non-stimulated (NWS) and stimulated (SWS) whole saliva, plasma, and erythrocytes, with Student’s t-test and Mann–Whitney U-test used for statistical analysis. We demonstrated that the activities of superoxide dismutase, catalase, and peroxidase were significantly higher in NWS, SWS, and erythrocytes of children with hypertension, similar to oxidative damage in proteins (advanced glycation end products) and lipids (malondialdehyde) as well as nitrosative stress markers (peroxynitrite and nitrotyrosine). The level of uric acid (UA) was significantly higher in NWS, SWS, and plasma of children with hypertension. UA concentration in SWS correlated positively with systolic and diastolic blood pressure and UA content in plasma. This parameter differentiates children with hypertension from healthy controls (AUC = 0.98) with a high degree of sensitivity (94%) and specificity (94%). Stimulated salivary flow was significantly lower in the hypertension group, similar to total protein content and salivary amylase activity. In summary, childhood hypertension is associated with hyposalivation as well as disturbances in antioxidant defense and enhanced oxidative/nitrosative damage both in the plasma/erythrocytes as well as saliva. Salivary UA may be a potential biomarker of hypertension in children.

## 1. Introduction

Hypertension occurs in over 25% of the world population [1]. More and more often the disease is also diagnosed in children. The prevalence of hypertension in people under 18 years of age is about 5%, and in most cases it is a secondary disease [2,3]. Indeed, as evidenced by epidemiological studies, hypertension in children is mainly associated with disorders of kidney parenchyma, endocrinopathy and heart defects. However, with the increasing incidence of obesity and insulin resistance, children are more and more often diagnosed with primary hypertension (particularly teenagers) [2,3,4].

Arterial hypertension is one of the main risk factors of cardiovascular diseases. According to the World Health Organization (WHO), hypertension is also the second-leading cause of death among all cardiovascular disease risk factors [5]. However, in the developmental age, hypertension rarely reveals any clinical symptoms [4].

A key role in the pathogenesis of hypertension is attributed to oxidative stress [6,7,8]. It has been demonstrated that overproduction of reactive oxygen (ROS) and nitrogen (RNS) species in endothelial cells is connected with excessive activity of NADPH (NOX) and xanthine (XO) oxidases as well as impaired activity of nitric oxide synthase (NOS), which facilitates oxidative damage to proteins and lipids and thus leads to an increase of peripheral resistance and pressure [6,7]. Indeed, the reduction of nitric oxide (NO) bioavailability seems to be of particular importance in the development of hypertension. It is well known that NO exerts vasodilating and anti-aggregation effects and inhibits the proliferation of vascular myocytes. In the conditions of its deficiency, the endothelial vasoconstrictive and pro-aggregation factors (produced in the cyclooxygenase pathway) gain the advantage [6,7,8,9]. Oxygen free radicals are also mediators of angiotensin II, endothelin and bradykinin, in this way participating in the remodeling of endothelial vascular walls [7,8]. Interestingly, angiotensin II-mediated oxidative stress (via enhanced NOX activity) is a major contributor to increased blood pressure and tissue inflammation [6,9]. Given the significant effect of oxidative/nitrosative stress on the development of hypertension, it is postulated that we can use redox biomarkers in the diagnostics and monitoring of treatment effectiveness [9]. Uric acid (UA), which is an independent risk factor for metabolic syndromes, hypertension, cardiovascular death, and chronic kidney disease, is of particular interest in modern laboratory medicine [9,10,11]. Although UA is the most important plasma antioxidant (accounting for about 70% of its antioxidant capacity), this compound can also generate free radicals, among other substances, by reaction with peroxynitrite. In addition, UA inhibits the activity of neuronal NOX in the juxtaglomerular apparatus, thereby reducing NO production in the kidneys [6,7,8,9].

Saliva is becoming an increasingly popular body fluid in laboratory medicine [12,13,14,15]. It is a non-invasive diagnostic tool, the collection of which is particularly convenient for children and teenagers [13,14]. It is believed that reducing a patient’s anxiety associated with the collection of the material for assays may encourage more frequent monitoring of health conditions and diagnosis of diseases at an early stage [16]. The clinical usefulness of biomarkers measured in saliva has been confirmed in children with diabetes and obesity [17,18,19] as well as in children with chronic kidney disease [16,20]. However, there are no reports on salivary redox biomarkers in children with hypertension. Similarly, the secretory function of salivary glands in hypertensive children has not been assessed so far. It is highly probable that, as in other oxidative stress-related diseases, the function of salivary glands as well as protein secretion in saliva are disturbed [21,22,23]. Because redox homeostasis cannot be characterized by a single biomarker, the aim of our study was to evaluate the salivary antioxidative barrier, the oxidative and nitrosative damage to proteins and lipids, as well as the secretory activity of salivary glands in children with hypertension compared to the controls. An important part of the study was also the search for salivary–blood correlations, and the assessment of the diagnostic utility of the assayed biomarkers.

## 2. Material and Methods

### 2.1. Ethical Issues

The research project was approved by the Local Bioethics Committee in Bialystok (permission number R-I-002/43/2018). It was implemented in accordance with the Declaration of Helsinki that defines procedures in human biomedical research. All subjects and/or their legal guardians gave their written consent to participate in the experiment.

### 2.2. Patients

The hypertension group included 53 children (28 boys, 25 girls) with arterial hypertension, treated in the Department of Pediatrics and Nephrology of the Medical University of Bialystok Children’s Clinical Hospital (Table 1). Samples for testing were taken between January 2018 and January 2019. The assessment of patient health was based on medical history, physical examination and laboratory and imaging results. The causes of hypertension were kidney diseases (47%) and primary hypertension (spontaneous) (53%). Patients with secondary hypertension presented with reflux nephropathy (44%), obstructive uropathy (20%), polycystic kidneys (16%), renal dysplasia (12%), and history of hemolytic–uremic syndrome (8%).

In our study, only 2 patients (3.8%) presented with high values of total cholesterol (TC), LDL-cholesterol, and triglycerides (TG); 1 studied patient (1.9%) had only high TC levels; two more patients (3.8%) had above normal LDL cholesterol; 2 more patients (3.8%) had high TG levels, giving a prevalence of dyslipidemia of 13.2%.

Hypertension was defined as the average value of the systolic (SBP) and/or diastolic (DBP) blood pressure ≥ 95th percentile for age, sex, and height measured on three or more occasions. Office blood pressure was measured using an automated oscillometric device (Datascope Accutor Plus) that had been validated for use in children. Four cuff sizes were available (child’s cuff, small adult cuff, adult cuff, and large adult cuff). The appropriate cuff size (bladder width at least 40% of arm circumference and length 80–100% of arm circumference) was determined by measuring the mid-upper arm circumference. Blood pressure (BP) was measured in the non-dominant arm in triplicate at 3 min intervals after a 5–10 min rest in the sitting position with the arm and back supported. The average values of the second and third measurements were used for subsequent analyses. Elevated blood pressure suspected on the basis of oscillometric readings was confirmed with measurements obtained by auscultation. All measurements were taken by trained staff.

Ambulatory blood pressure monitoring (ABPM) was used for confirmation of hypertension in children and adolescents with elevated office blood pressure measurements [24]. ABPM was performed using the oscillometric Boso TM-2430 PC2. The monitors were programmed to measure BP every 15 min during waking hours (8:00–22:00) and every 30 min during sleeping hours (22:00–8.00). The periods were corrected according to the patient’s diaries. Readings with a minimum 80% of measurement and without a break longer than 2 h were considered sufficient. The mean SBP and DBP were calculated separately for the 24 h and for the awake and asleep periods. Additionally, systolic blood pressure load (SBPL) and diastolic blood pressure load (DBPL) during the day and night were analyzed. SBPL and DBPL were calculated as a percentage of the frequency with which they exceeded the upper level of the normal range during each time bin to the total frequency during measurement of BP for the same time. Using the least median squares (LMS) method, sex-specific L, M, and S reference values were calculated for 24 h and daytime and nighttime mean values of SBP and DBP. The LMS values were taken from the OLAF study published by Kulaga et al. [25]. Hypertension was defined as a mean systolic BP level ≥ 95th percentile (1.5 SDS) and SBPL or DBPL load of more than 25% [24,26,27].

Patients who met all the following inclusion criteria were enrolled into the hypertensive group: (1) age 6–18 years, (2) arterial hypertension defined as either systolic and/or diastolic BP ≥ 95th percentile measured on three or more occasions and verified by ABPM as mean daytime and nighttime systolic BP (SBP) levels of ≥ 95th percentile for age, sex and height, and a load SBP or DBP of > 25% [24,26,27,28], (3) normal clinical examination, (4) no clinical and laboratory signs of infection, (5) normal levels of cortisol and thyroid-stimulating hormone, (6) normal electrocardiogram, and (7) signed informed consent.

None of our patients presented with target organ damage as we performed ECG, ophtalmological complete eye assessment, and assessment of UACR (urinary albumin/creatine ratio) (data not shown). Albuminuria was diagnosed if UACR ranged between 30 and 300 mg/g creatinine.

The control group consisted of 53 normotensive children attending the Specialist Dental Clinic (Department of Pediatric Dentistry) of the Medical University of Bialystok for regular check-ups. Samples for testing were collected after the study group was assembled between January 2019 and October 2019. Children in the control group were qualified on the basis of medical history and screening tests. Office blood pressure was measured similarly to children with hypertension. The control was matched by sex and age (± 3 months) to the hypertension group.

The exclusion criteria in the cases and the control group were a history of heart failure, hepatic dysfunction, the presence of systemic diseases: metabolic (type 1 and 2 diabetes, insulin resistance), autoimmune (lupus erythematosus, systemic sclerosis, arthritis, ulcerative colitis, thyroiditis), cancerous, infectious, gastrointestinal and pulmonary diseases, clinical or laboratory signs of secondary hypertension (due to thyroid or heart disease, abnormal Doppler of the renal arteries), and/or target organ damage (documented left ventricular hypertrophy, hypertensive retinopathy or increased urinary albumin/creatinine ratio). Children with acute infections were also eliminated from the study. Patients with a history of oral contraceptive use, current therapy with medications known to affect serum uric acid levels (e.g., allopurinol and febuxostat) and blood pressure values (other than hypertensive drugs) were excluded from the study.

It is widely believed that the use of 3 or more drugs significantly increases the incidence of xerostomia/hyposalivation [29,30]. Therefore, cases taking more than two antihypertensive drugs were not included in the study. Additionally, patients taking antibiotics, glucocorticosteroids, non-steroidal anti-inflammatory drugs, vitamins, and dietary supplements for at least 3 months before saliva collection were excluded from the study, similarly to individuals with poor oral hygiene and/or gingivitis (see: dental examination).

In all patients, BMI was calculated as weight (kg) divided by the square of height (m^2^). BMI z-scores that reflect the standard deviation score (SD) for age- and sex-appropriate BMI distribution were calculated according to the LMS method [31], using reference values from a WHO study [32]. Based on the international norms from WHO for age- (with an accuracy of 1 month) and sex-specific BMI, BMI cut-offs for children over 5 years of age were the following: obesity, BMI z-score ≥ + 2 SD [32].

### 2.3. Saliva Collection

The research material was mixed non-stimulated (NWS) and stimulated (SWS) whole saliva. All children and their legal guardians were thoroughly familiarized with the saliva collection procedure prior to the study. Saliva was collected from children who were not physically active for the last 24 h, after an all-night rest, always between 7 and 9 a.m. Individuals from the hypertension and the control group did not take any medicines for at least 8 h prior to saliva collection. In addition, patients did not consume any meals or drinks (other than water), and refrained from performing any oral hygiene procedures (brushing their teeth, chewing gum, etc.) at least 2 h before saliva collection. Immediately before saliva sampling, a detailed interview was conducted with the patient and their parents. Children who did not meet the protocol criteria were eliminated from the study. All of the examinations were performed by the same experienced pediatric dentist (J.S.).

We used the saliva collection protocol described in detail earlier [16,20,33]. Briefly, saliva was taken from all children by spitting into a sterile Falcon-type tube placed in an ice container. The oral cavity was rinsed twice with distilled water at room temperature before the beginning of saliva collection. Saliva was taken in a sitting position, with the patient’s head slightly inclined downwards (with minimized facial and lip movements), always in the same child-friendly room, upon at least 5 min of adaptation to the environment. The saliva collected during the first minute was discarded. The time of NWS collection was 15 min. SWS was collected similarly to NWS, and saliva was stimulated by dropping 10 µl of citric acid (2%, *w/w*) on the tip of the tongue every 30 s. The time of SWS collection was 5 min [16,20,33].

Immediately after collection, the volume of saliva was measured with a pipette set to 100 µL. Then, saliva was immediately centrifuged (20 min, 4 °C, 3000× *g*; MPW 351, MPW Med. Instruments, Warsaw, Poland) and the supernatant fluid was preserved for assays. To protect the samples against oxidation, butylated hydroxytoluene (BHT, Sigma-Aldrich, Germany) was added to the obtained supernatants in the amount of 10 μL 0.5 M BHT in acetonitrile (ACN)/1 mL saliva [16,20,33]. Blood contamination was not observed in any of the samples. The samples were frozen at −80 °C and they were not stored for longer than six months.

The minute flows of NWS and SWS were calculated by dividing the volume of saliva by the time necessary for its secretion (mL/min). NWS flow below 0.2 mL/min and SWS flow below 0.9 mL/min were considered as decreased salivary secretion (hyposalivation/salivary hypofunction) [16,20,33,34].

To assess the salivary gland function, the activity of salivary amylase was also determined. A colorimetric method with 3,5-dinitrosalicylic acid was used and absorbance was measured at 540 nm [35]. The activity of salivary amylase was performed in triplicates.

### 2.4. Dental Examination

After saliva collection, a clinical dental examination in artificial lighting (10,000 lux) was performed. According to the WHO criteria [36], a mirror, an explorer and a periodontal probe were used. The incidence of caries was determined using dmft index (decay, missing, filled teeth) which is the sum of teeth with caries (D), teeth extracted because of caries (M), and teeth filled because of caries (F). This parameter was calculated for permanent teeth (DMFT) and milk teeth (dmft). API (approximal plaque index) according to Lange was used to assess the status of oral hygiene. API determines the percentage of tooth surface with plaque. SBI (Sulcus Bleeding Index) according to Muhemann and Son, and GI (Gingival Index) according to Löe and Silness were used to assess the condition of gums. SBI showed the intensity of bleeding from the gingival sulcus after probing, while GI criteria included qualitative changes in the gingiva [36].

Because the main source of salivary oxidative stress are periodontal disease and dental caries [20,33,37], children with poor oral hygiene (API > 20) and gingivitis (SBI > 0.5, GI > 0.5) were excluded from the experiment. All of the dental examinations were performed by the same dentist (J.S.). In 20 patients, the inter-rater agreements between the examiner (J.S.) and another experienced pediatric dentist (A.Z.) were assessed. The reliability for all parameters was > 0.98.

### 2.5. Blood Collection

Whole blood was taken from fasting patients after an all-night rest, always between 7 and 8 a.m. We used the S-Monovette^®®^ K3 EDTA blood collection system (Sarstedt, Nümbrecht, Germany), and samples were immediately centrifuged (1500× *g*; 4 °C, 10 min) [27]. Hemolysis was not observed in any sample. Supernatant fluid (plasma) was preserved for further studies, while erythrocytes were rinsed three times with a cold solution of 0.9% NaCl, and then hemolyzed by adding 9 volumes of cold phosphate buffer (50 mM, pH 7.4). Similarly to saliva, BHT (10 μL 0.5 M BHT/1 mL sample) was added to blood samples that were then frozen at −80°C [33].

A second portion of blood was used for routine laboratory tests. All patients underwent morphological (leukocytes, erythrocytes, hemoglobin, hematocrit, platelets) and biochemical (creatinine, urea, HDL, LDL, total cholesterol, glucose) examinations. The tests were performed in the Laboratory of Pediatric Diagnostics of the Medical University of Bialystok Children’s Clinical Hospital with the use of automated blood analyzers (Sysmex XN1000 and Abbott Architect c8000).

Dyslipidemia in studied children was defined as an abnormal lipid profile value in at least one of: LDL-cholesterol (≥ 130 mg/dL), total cholesterol (TC) (≥ 200 mg/dL), HDL-cholesterol (< 40 mg/dL) and/or triglycerides (TG) (in children 0–9 years ≥ 100 mg/dL, and in those aged 10–19yrs ≥ 130 mg/dL) [38,39,40].

The estimated glomerular filtration rate (eGFR) was calculated using the Schwartz formula—eGFR (mL/min/1.73 m^2^) = 0.413 × [height in cm/sCr], where sCr is the level of creatinine in the serum [41].

The concentration of plasma interleukin-6 (IL-6) was determined in duplicate samples using enzyme-linked immunosorbent assay (ELISA). Commercial kits from EIAab Science Inc. Wuhan (Wuhan, China) were used, according to the manufacturer’s instructions. The absorbance was measured at 450 nm using an Infinite M200 PRO Multimode Microplate Reader Tecan.

### 2.6. Total Protein Assay

The total protein content was determined with the bicinchoninic acid (BCA) method, using a commercial kit (Thermo Scientific PIERCE BCA Protein Assay (Rockford, IL, USA)). Bovine serum albumin (BSA) was used as a standard.

### 2.7. Redox Assays

The activity of antioxidant enzymes was assessed in NWS, SWS and erythrocytes, while the concentration of non-enzymatic antioxidants, redox status, and the content of oxidative and nitrosative stress products were assayed in NWS, SWS, and blood plasma. All determinations were performed in duplicate samples, and absorbance/fluorescence of the samples was measured with an Infinite M200 PRO Multimode Microplate Reader Tecan. The results were standardized to 1 mg of total protein.

### 2.8. Enzymatic and Non-Enzymatic Antioxidants

The activity of superoxide dismutase (SOD; E.C. 1.15.1.1) was determined by the Misra and Fridovich method [42], following the absorbance changes accompanying adrenaline oxidation at 480 nm wavelength. It was assumed that 1 SOD unit corresponds to 50% of the inhibition of adrenaline self-oxidation to adrenochrome. The activity of SOD was expressed in mU/mg protein.

Catalase activity (CAT; EC 1.11.1.6) was determined spectrophotometrically according to Aebi [43] by measuring the rate of hydrogen peroxide decomposition compared to the blank sample at 240 nm wavelength. 1 CAT unit was defined as the amount of the enzyme needed to decompose 1 mmol of hydrogen peroxide within 1 min. CAT activity was expressed in nmol H_2_O_2_/min/mg protein.

The activity of salivary peroxidase (Px; EC 1.11.1.7) was measured spectrophotometrically according to Mansson-Rahemtulla et al. [44]. We evaluated absorbance changes accompanying the reduction of 5,5′-dithiobis-(2-nitrobenzoic acid) (DTNB) to 5-thio-2-nitrobenzoic acid (TNB) at 412 nm. The activity of Px was expressed in mU/mg protein.

The activity of erythrocyte glutathione peroxidase (GPx; EC 1.11.1.9) was determined spectrophotometrically based on the conversion of NADPH (reduced nicotinamide adenine dinucleotide) to NADP^+^ (nicotinamide adenine dinucleotide cation) at 340 nm wavelength [45]. It was assumed that 1 unit of GPx catalyzes the oxidation of one millimole of NADPH for one minute. The activity of GPx was expressed in mU/mg protein.

The concentration of reduced glutathione (GSH) was determined spectrophotometrically based on the reduction of DTNB to 2-nitro-5-mercaptobenzoic acid at 412 nm wavelength [46]. The concentration of GSH was expressed in µg/mg protein.

Uric acid (UA) concentration was determined spectrophotometrically by measuring the absorbance of 2,4,6-tripyridyl-s-triazine complex with iron ions and UA present in the examined sample at 490 nm wavelength. We used a commercial reagent kit (QuantiChromTMUric Acid Assay Kit DIUA-250; BioAssay Systems, Hayward, CA, USA). The concentration of UA was expressed in µg/mg protein.

### 2.9. Redox Status

Total antioxidant capacity (TAC) was determined by the spectrophotometric method, measuring changes in the absorbance of ABTS^*+^ solution (3-ethylbenzothiazoline-6-sulfonic acid radical cation) at 660 nm wavelength [47]. TAC was calculated from the standard curve for Trolox (6-hydroxy-2,5,7,8-tetramethylchroman-2-carboxylic acid) and expressed in µmol/mg protein.

Total oxidant status (TOS) was determined spectrophotometrically according to Erel [48]. In the presence of oxidants contained in the sample, Fe^2+^ ions were oxidized to Fe^3+^ which then formed a colored complex with xylenol orange. The level of TOS was calculated from the standard curve for hydrogen peroxide and expressed in nmol H_2_O_2_ equiv/mg protein.

The oxidative stress index (OSI) was calculated as the quotient of TOS to TAC [49].

### 2.10. Oxidative Stress Products

The level of advanced glycation end products (AGE) of proteins was determined spectrofluorimetrically according to Kalousová et al. [50]. The samples were diluted in 0.02 M PBS buffer (1:5, *v/v*) [51] and fluorescence was measured at 350 nm excitation wavelength and 440 nm emission wavelength. AGE content was expressed in arbitrary fluorescence units (AFU)/mg of total protein.

The concentration of malondialdehyde (MDA) was determined spectrophotometrically using thiobarbituric acid (TBA) [52]. The absorbance of samples was measured at 535 nm wavelength, and 1,1,3,3-tetraethoxypropane was used as standard. MDA concentration was expressed in µmol/mg protein.

### 2.11. Nitrosative Stress Products

The concentration of nitric oxide (NO) was assessed colorimetrically using sulfanilamide and N-(1-naphthyl)-ethylenediamine dihydrochloride [53,54]. The absorbance was measured at 490 nm wavelength. NO concentration was expressed in nmol/mg protein.

The level of peroxynitrite was measured colorimetrically based on peroxynitrite-mediated nitration resulting in the formation of nitrophenol [55]. The absorbance was measured at 320 nm wavelength.

Nitrotyrosine concentration was determined by the ELISA method, using a commercial diagnostic kit (Immundiagnostik AG; Bensheim, Germany) and expressed in pmol/mg protein.

### 2.12. Statistical Analysis

The Shapiro–Wilk test was used to determine the normality of distribution, and the Student’s t-test was used to compare the hypertension group with the controls. Where the data distribution was not normal, the Mann–Whitney U-test was used. The two-sided *p*-value was used. The value of *p* < 0.05 was considered statistically significant. Multiplicity-adjusted p value was also calculated. The results were presented as an arithmetic mean ± standard deviation (SD). Because the vast majority of data showed a normal distribution, the Pearson correlation coefficient was used. Receiver Operating Characteristic (ROC analysis) was used to assess the diagnostic utility of the examined biomarkers. For this purpose, ROC curves were generated, and then the area under the curve (AUC) was calculated. Optimal cut-off values were determined for each parameter that ensured high sensitivity with high specificity. Statistical analysis was performed using GraphPad Prism 8 for Mac (GraphPad Software, La Jolla, USA).

The number of patients was set a priori based on our pilot study involving 15 patients. Online sample size calculator (ClinCalc) was used. Variables used for sample size calculation were NWS and SWS flow rate, activity/concentration of some antioxidant enzymes (SOD, CAT, UA), and concentration of oxidative damage products (AGE, MDA). The level of significance was set at 0.05 and power of study was 0.9. The ClinCalc software provided the sample size for one group. The minimum number of patients was 32.

## 3. Results

### 3.1. Clinical Characteristics

Clinical characteristics of the subjects are presented in Table 1.

Ambulatory blood pressure monitoring (ABPM) values of children with hypertension are presented in Table 2.

### 3.2. Salivary Gland Function and Dental Examination

Salivary flow rate, total protein content, and α-amylase activity were significantly lower in SWS of hypertensive children compared to controls. Importantly, SWS secretion was below the reference values (< 0.9 mL/min) (Table 3).

We have not found any significant differences in the incidence of dental caries as well as oral hygiene between the control and hypertension group (Table 3).

### 3.3. Enzymatic and Non-Enzymatic Antioxidants

**Figure 1:**
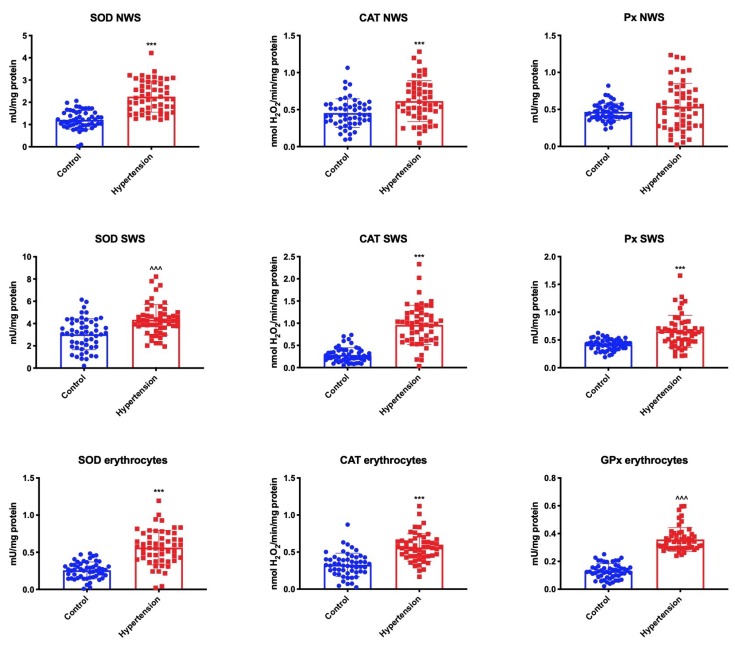
Antioxidant enzymes in children with hypertension and healthy controls. CAT—catalase; GPx—glutathione peroxidase; NWS—non-stimulated whole saliva; Px—salivary peroxidase; SOD—superoxide dismutase; SWS—stimulated whole saliva. Differences statistically significant at: *** *p* < 0.001 (Student’s t-test); ^^^^^ *p* < 0.001 (Mann–Whitney U-test).

To assess the antioxidant barrier, we used both antioxidant enzymes (SOD, CAT, Px/GPx) and non-enzymatic antioxidants (GSH and UA). SOD catalyzes the reaction of superoxide anion dismutation to oxygen and hydrogen peroxide which is then decomposed in the presence of CAT and peroxidases (Px/GPx). We observed significantly higher SOD activity in NWS, SWS, and erythrocytes of children with hypertension compared to the control group. Similarly, CAT activity in NWS, SWS and erythrocytes, and Px activity in SWS and blood, were considerably higher in children from the hypertension group (Figure 1).

**Figure 2:**
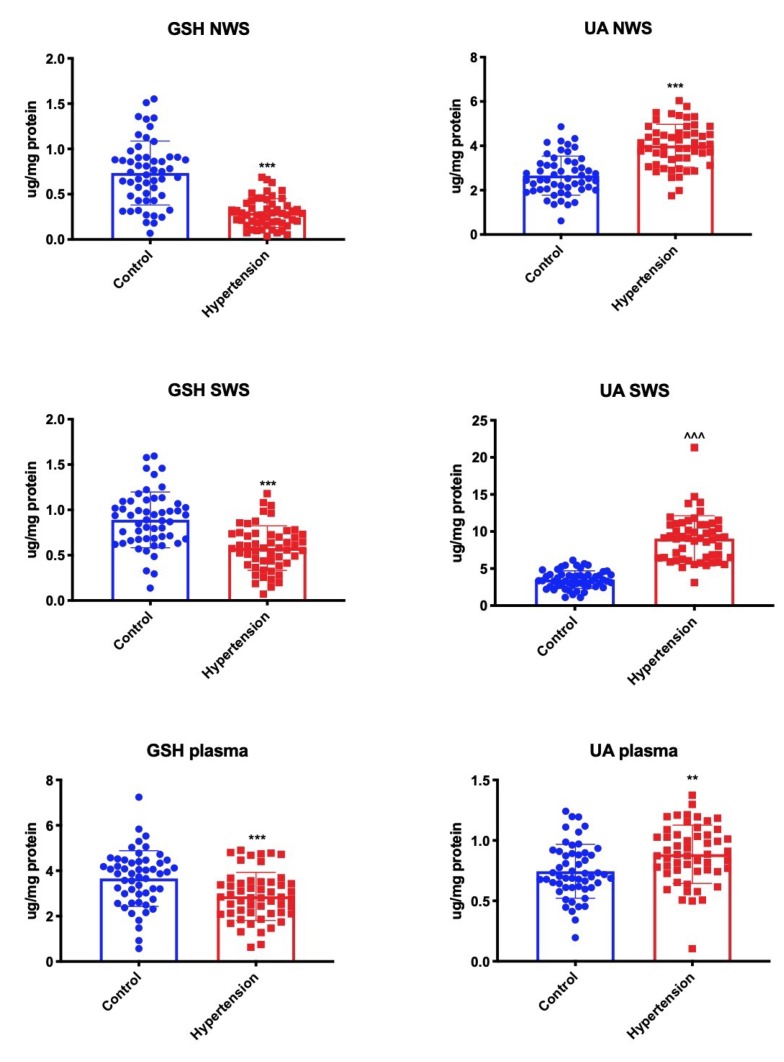
Non-enzymatic antioxidants in children with hypertension and healthy controls. GSH—reduced glutathione; NWS—non-stimulated whole saliva; SWS—stimulated whole saliva; UA—uric acid. Differences statistically significant at: ** *p* < 0.01, *** *p* < 0.001 (Student’s t-test); ^^^^^ *p* < 0.001 (Mann–Whitney U-test).

UA concentration in non-stimulated saliva, stimulated saliva, and plasma was significantly higher in children with hypertension compared to the controls. However, we demonstrated a significant decrease in GSH levels in both NWS, SWS, and plasma of children with hypertension compared to healthy subjects (Figure 2).

### 3.4. Redox Status

**Figure 3:**
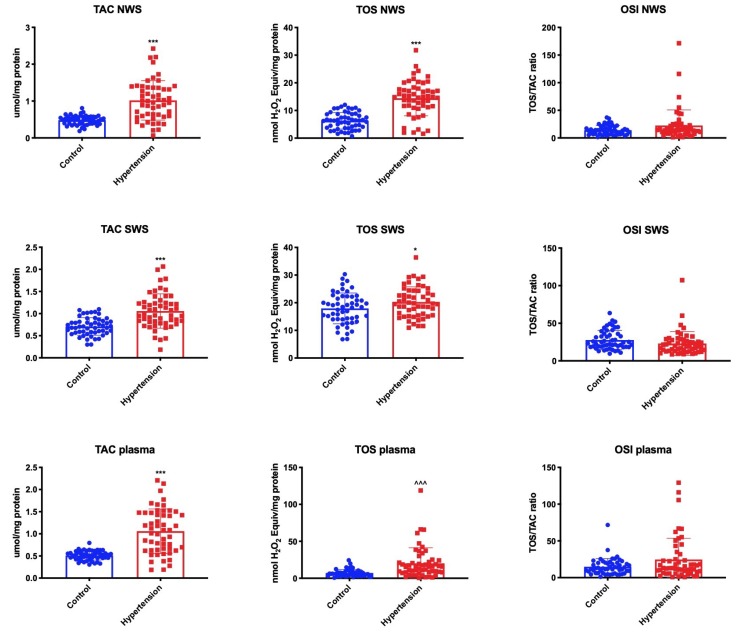
Redox status in children with hypertension and healthy controls. NWS —non-stimulated whole saliva; OSI—oxidative stress index; SWS—stimulated whole saliva; TAC—total antioxidant capacity; TOS—total oxidant status. Differences statistically significant at: * *p* < 0.05, *** *p* < 0.001 (Student’s t-test); ^^^^^ *p* < 0.001 (Mann–Whitney U-test).

The redox status of saliva and plasma were assessed by measuring total antioxidant capacity (TAC) and total oxidant status (TOS). The oxidative stress index (OSI) was also calculated as the quotient of TOS to TAC. The TAC level was significantly higher in NWS, SWS and plasma of children with hypertension, similar to the TOS level in saliva and blood. However, the oxidative stress index did not differ significantly between the hypertension group and healthy controls (in NWS, SWS, and plasma) (Figure 3).

### 3.5. Oxidative Stress Products

**Figure 4:**
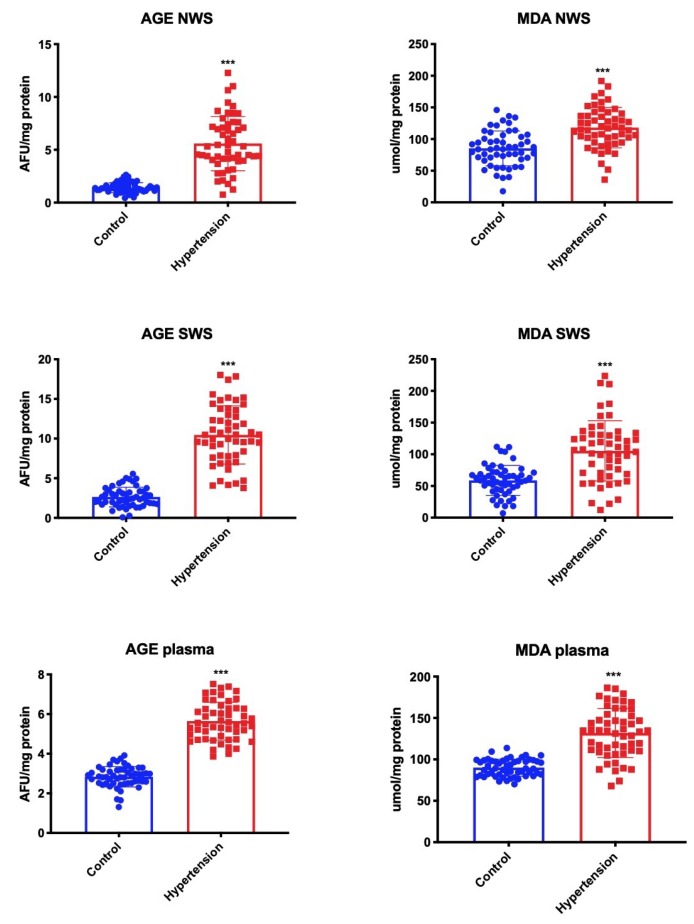
Oxidative stress products in children with hypertension and healthy controls. AGE—advanced glycation end products; MDA—malondialdehyde; NWS—non-stimulated whole saliva; SWS—stimulated whole saliva. Differences statistically significant at: *** *p* < 0.001 (Student’s t-test).

We assessed the severity of oxidative stress based on oxidative damage to proteins (AGE, carbonyl stress marker) and lipids (MDA, lipoperoxidation product). We observed significantly higher AGE content in NWS, SWS, and plasma of children with hypertension compared to the control group. We also noted considerably higher concentration of MDA in both types of saliva as well as in plasma of children from the hypertension group (Figure 4).

### 3.6. Nitrosative Stress

**Figure 5:**
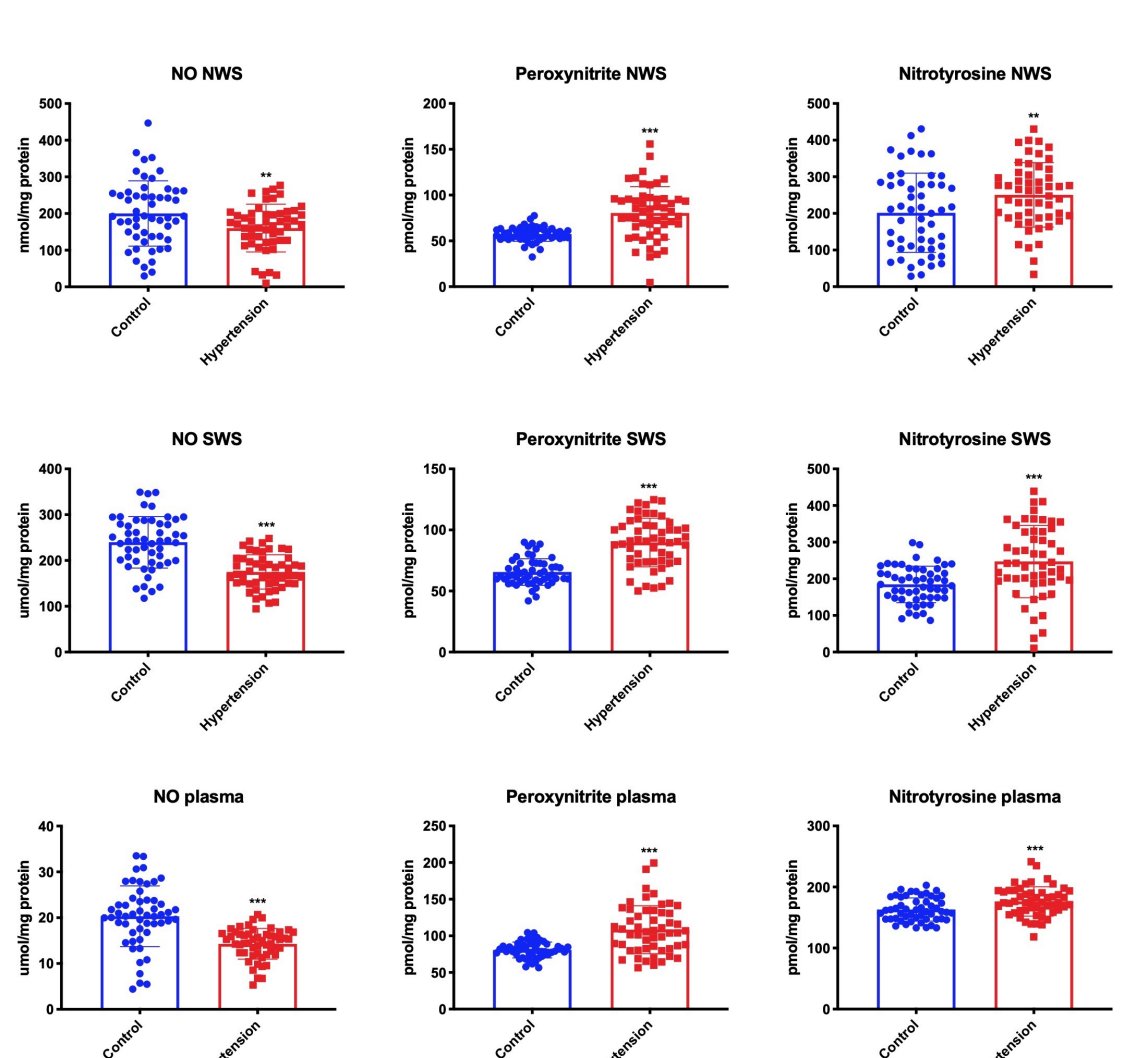
Nitrosative stress in children with hypertension and healthy controls. NO—nitric oxide; NWS—non-stimulated whole saliva; SWS—stimulated whole saliva. Differences statistically significant at: ** *p* < 0.01, *** *p* < 0.001 (Student’s t-test).

Nitrosative stress was assessed by measuring the levels of NO, peroxynitrite (the product of NO and superoxide anion reaction), and nitrotyrosine (marker of nitrosative damage to proteins). We demonstrated a significantly lower concentration of NO in NWS, SWS, and plasma in children from the hypertension group, while the levels of peroxynitrite and nitrotyrosine were considerably higher in NWS, SWS, and plasma of children with hypertension vs. the control group (Figure 5).

### 3.7. Correlations

Statistically significant correlations are presented in Table 4 (control group) and Table 5 (hypertension group).

In the control group, CAT activity correlates with SOD activity in stimulated whole saliva. The concentration of protein glycooxidation products (AGE) and lipid oxidation products (MDA) in NWS correlated with their levels in plasma (Table 4).

In NWS of children with hypertension we observed a positive correlation between SOD activity and TOS level. Moreover, TOS correlated positively with CAT and UA in SWS of children with hypertension. We also observed a positive correlation of UA measured in SWS and blood pressure (SBP and DBP) in children with hypertension. Interestingly, salivary AGE and MDA also correlated positively with SBP and DBP (both in NWS and SWS) (Table 5).

We demonstrated a negative correlation between the concentration of peroxynitrite and GSH in SWS of children with hypertension as well as a positive correlation between peroxynitrite and nitrotyrosine. The content of peroxynitrite in SWS also correlated positively with the content of MDA.

We observed a negative correlation between the SWS minute flow and MDA, peroxynitrite, and nitrotyrosine, and a positive correlation with NO measured in SWS (Table 5).

As in the control group, we showed a strong correlation between AGE and MDA concentration in non-stimulated saliva and their plasma content (Table 5).

### 3.8. ROC Analysis

The results of ROC analysis are presented in Table 6 and Table 7.

A large proportion of the redox biomarkers determined in NWS, SWS, erythrocytes, and plasma significantly differentiated children with hypertension from the age- and sex-matched group of healthy controls (Table 6 and Table 7). Particularly noteworthy is UA content measured in SWS (AUC = 0.98), which, at sensitivity = 94% and specificity = 96%, distinguished the hypertension group from the controls (Table 6). Moreover, AGE measured in NWS, SWS, and plasma also presented high diagnostic value in recognizing hypertension in children (Table 6 and Table 7).

## 4. Discussion

Our study was the first to assess salivary redox homeostasis in children with hypertension. We demonstrated that childhood hypertension is associated with abnormalities of the antioxidant barrier as well as oxidative damage to proteins and lipids both in saliva (NWS/SWS) and plasma/erythrocytes. Interestingly, in children with hypertension we observed reduced secretion of stimulated saliva. Salivary UA may be a potential biomarker of hypertension.

It is emphasized that oxidative stress plays a key role in the pathogenesis of hypertension [6,7,8]. It has been proven that oxygen free radicals increase expression of angiotensin II receptor (AT1R), induce proinflammatory signaling pathways (such as NF-E2-related factor 2, Nrf2, nuclear factor-κB, NF-kB and activator protein-1, AP1) and activate genes responsible for angiogenesis and proliferation of endothelial cells [6,8,56]. Additionally, ROS and RNS impair the relaxation phase of smooth muscle tissue inside blood vessels, and increase endothelial permeability to lipoproteins that, when in oxidized form (mainly as oxLDL), intensify inflammation [6,56].

It is well known that the assessment of oxidative stress level cannot be based solely on a few redox biomarkers [57]. In our study, we evaluated the enzymatic and non-enzymatic antioxidant barrier, redox status, oxidative damage to proteins and lipids, as well as nitrosative stress products. We also assessed the diagnostic usefulness of the analyzed redox biomarkers using ROC analysis.

Antioxidants are the first line of defense against oxidative stress. In our study, we observed increased activity of antioxidant enzymes (↑ SOD, ↑ CAT, ↑ Px/GPx) in NWS, SWS, and erythrocytes in children with hypertension compared to the controls. The content of UA and TAC was significantly higher in the saliva and blood of children with hypertension, which suggests an adaptive response of the body to boosted production of ROS and RNS. Although we did not evaluate the rate of free radical production directly, our hypothesis may be confirmed by a positive correlation between the concentration/activity of antioxidants (SOD, CAT, UA) and TOS levels in children with hypertension. The total oxidant status is believed to reflect the total content of oxidants in the biological system [48,57]. As shown in in vitro as well as in vivo studies, the main source of ROS in hypertension is overexpression of pro-oxidant enzymes (NOX and XO) generating considerable amounts of superoxide radical anion (O_2_^∙−^) and hydrogen peroxide (H_2_O_2_) [6,8,56]. It is not surprising that we observed increased activity of SOD (enzyme that transforms O_2_^∙−^) and CAT (enzyme decomposing H_2_O_2_). It is noteworthy that XO catalyzes the oxidation reaction of hypoxanthine to xanthine and uric acid [58], which may also explain increased concentration of UA in our patients. Although UA is considered the most important salivary antioxidant (accounting for 70–80% of salivary antioxidant capacity) [37,59], this compound has also a strong pro-oxidant effect. However, what role does UA play in this scenario? Although our study does not exactly explain this, the pro-oxidative and pro-inflammatory activity of UA could be demonstrated by a positive correlation of UA and TOS in SWS, as well as UA in SWS and plasma IL-6 in the cases. Indeed, UA has been proven to generate oxygen and nitrogen free radicals, block NOS activity and promote the formation of oxLDL [58,60]. Interestingly, this phenomenon is particularly common during reperfusion. UA has been shown to react with peroxynitrite to generate an amino carbonyl radical capable of alkylating biomolecules. However, under oxidative stress conditions, uric acid can also be rapidly degraded and its by-products are very cytotoxic. In the presence of pro-oxidant metal ions (especially Cu^2+^ and Fe^2+^), UA can also intensify lipid peroxidation [58,60]. Since our study does not explain the role of uric acid in organ complications of hypertension, further studies and clinical observations are necessary. Nevertheless, in hypertensive children, we observed a positive correlation of salivary UA with DBP, SBP, and DBP/SBP 24 h Z-score.

Despite the strengthened antioxidant barrier (↑ SOD, ↑ CAT, ↑ Px/GPx, ↑ UA, ↑ TAC), in children with hypertension we observed an increase in oxidative damage to proteins (↑ AGE, AOPP) and lipids (↑ MDA), not only at the central level (plasma), but also in NWS and SWS. It is believed that the products of oxidative modifications may penetrate through the damaged endothelium into the walls of blood vessels and then be captured by macrophages, resulting in the formation of foam cells [6,61]. Under these conditions, NF-kB signaling pathway is activated, which induces the production of proinflammatory cytokines (IL-2, IL-6), chemokines (monocyte chemoattractant protein-1—MCP-1), and adhesion molecules (intercellular adhesion molecule-1, ICAM-1, vascular cell adhesion protein 1, VCAM-1) [6,7,61]. Interestingly, both protein glycooxidation (AGE) and lipid peroxidation products (MDA) correlated positively with SBP and DBP. Moreover, the level of nitrosative stress was significantly higher in NWS, SWS, and plasma of children with hypertension compared to the controls (↑ peroxynitrite, ↑ nitrotyrosine).

Proper function of blood vessels depends mainly on the activity of endothelial cells. It synthesizes numerous substances, the most important vasodilator of which is nitric oxide (NO). Interestingly, we found a decrease in NO concentration in plasma as well as NWS and SWS of children with hypertension compared to the controls. It is believed that the basic mechanism reducing the bioavailability of NO is the direct reaction of nitric oxide with O_2_^∙−^, resulting in the formation of highly reactive peroxynitrite with strong oxidizing properties [58,62]. Indeed, it has been demonstrated that peroxynitrite interferes with mitochondrial function and oxidizes thiol groups of enzymatic and signaling proteins [62]. Considering that glutathione is an important source of cellular thiols [63], these observations are confirmed by the negative correlation between the concentration of peroxynitrite and GSH in children with hypertension. This compound is also responsible for nitration of the phenolic groups of proteins (mainly tyrosine and tryptophan) [64], which can be evidenced by increased concentration of nitrotyrosine in children with hypertension, and a positive correlation between nitrotyrosine and peroxynitrite. Finally, peroxynitrite may also induce lipid peroxidation [58,62], as suggested by its positive correlation with MDA content. However, further research is required to clarify the role of nitrosative stress in maintaining oral homeostasis.

In addition to its vasodilatory effect, NO is an important signaling molecule secreted at the nerve endings of the parasympathetic nervous system. It is noteworthy that this compound participates in initiating saliva secretion [65,66]. It has been demonstrated that NO increases the concentration of Ca^2+^ ions in secretory cells of salivary glands by opening water channels (aquaporins) [65,66]. Thus, it is obvious that in children with hypertension we observed reduced SWS secretion and total protein content compared to the controls. Importantly, stimulated saliva secretion was below reference values (<0.9 mL/min) [16,20,33]. In addition, salivary amylase activity was also significantly lower in stimulated saliva of hypertensive children. It is well known that α-amylase is one of the most important salivary proteins. It is also a recognized biomarker of salivary gland damage [35,67]. During stimulation, over 60% of saliva is produced by parotid glands [33,59]. Therefore, children with hypertension suffer from hypofunction of these salivary glands. As a result of hyposalivation, the processes of enamel demineralization and the development of dental caries may intensify [20,65,67]. Thus, children with hypertension should receive additional dental care. Interestingly, we observed a negative correlation between SWS flow rate and MDA, peroxynitrite, and nitrotyrosine, as well as a positive correlation with NO. Unfortunately, our study does not explain the causal relationship between oxidative stress and hyposalivation. However, it can be assumed that, as in other oxidative-stress-related diseases, products of protein and lipid oxidation can accumulate/aggregate in salivary glands, leading to secretory cell damage and a decrease in SWS secretion [21,22,49,68]. Indeed, oxidative stress is a key pathological factor responsible for hyposalivation in the course of many systemic diseases. Its role in salivary hypofunction has been confirmed in patients with diabetes, insulin resistance, chronic kidney disease, psoriasis, and cancer [20,68,69,70].

Hypotensive drugs may also be responsible for reduced saliva secretion. Indeed, pharmacological treatment can affect sodium and potassium transporters in the secretory cells as well as change the electrolyte composition of saliva [4,67]. Moreover, the use of three or more drugs significantly increases the frequency of xerostomia and hyposalivation [29,30]. However, in our study we excluded children receiving more than two hypotensive drugs. Importantly, we did not show any significant differences in the analyzed redox parameters/salivary flow rate in children taking 1, 2 or no medications (data not shown).

An important part of our study was also the evaluation of the saliva–blood correlation coefficients as well as the diagnostic usefulness of salivary redox biomarkers. Salivary antioxidants did not correlate with the content of these substances in plasma/erythrocytes (except UA), which is not surprising because the oral cavity is the only place in the body exposed to numerous pro-oxidant factors. These include: xenobiotics (tobacco smoke, medicines, air pollution), food, dental treatment, and dental materials [37,59]. Thus, the salivary antioxidant barrier does not necessarily reflect the central redox status. However, in children with hypertension, the concentration of protein and lipid oxidation products in NWS correlates with their levels in plasma, indicating that saliva can be an alternative diagnostic body fluid to blood. Moreover, in ROC analysis we showed that numerous redox biomarkers with high sensitivity and specificity differentiate healthy children from those with hypertension. Particularly promising results were observed for UA measured in SWS (AUC = 0.98, sensitivity = 94.34%, specificity = 96.23%), which was further confirmed by the positive correlation with SBP and DBP. Interestingly, UA concentration in SWS reflects its content in plasma, which also proves the diagnostic utility of salivary UA. Indeed, more and more data suggest the participation of uric acid in the pathogenesis of metabolic and cardiovascular diseases [9,10,11]. Therefore, further studies are necessary to assess the clinical value of salivary UA in a larger population of children with hypertension. Saliva can be collected in a non-invasive and simple manner; the procedure is cheap and does not require any involvement of medical personnel. Non-invasive sampling is particularly important for young children. Thus, our study is the starting point for further basic and clinical research. Additionally, it is necessary to develop reference values for salivary redox biomarkers and to standardize saliva collection protocols.

Despite the undoubted advantages, our work has also many limitations. We only examined the selected biomarkers of oxidative/nitrosative stress; therefore, we cannot fully characterize salivary redox homeostasis in children with hypertension. In addition to the underlying disease, obesity and hypotensive drugs may also disturb the oxidation–reduction balance of the body. The imperfection of inclusion and exclusion criteria for the study, solely bivariate statistical analyses and lack of full periodontological examination are also limitations of our work. However, despite the relatively small hypertension group, it should be noted that the study was conducted on children from which non-stimulated/stimulated whole saliva, plasma, and erythrocytes were collected. Additionally, the study enrolled patients carefully selected for their comorbidities and periodontal status, which is undoubtedly the strength of the study.

## 5. Conclusions

1. Childhood hypertension is associated with disturbances in enzymatic and non-enzymatic antioxidant defense as well as enhanced oxidative and nitrosative damage both in the plasma/erythrocytes as well as salivary glands (NWS and SWS).
2. In hypertension, the secretion of stimulated saliva decreases. Children with hypertension should receive additional dental care.
3. Salivary UA may be a potential biomarker of hypertension in children. However, further studies are necessary to assess its diagnostic utility in a larger group of patients.

## Figures and Tables

**Table 1:** Clinical characteristics of children with hypertension and healthy controls.

		Control *n* = 53	Hypertension *n* = 53
General Data			
Age (years)		13 ± 3.1	13 ± 3.5
Male *n* (%)		28 (53%)	28 (53%)
BMI (kg/m^2^)		22 ± 2.6	26 ± 5.9 *
SDS BMI		0.8 ± 0.2	2.4 ± 0.5
Obesity *n* (%)		0 (0)	20 (38%)
Blood Pressure			
SBP (mmHg)		106 ± 10.5	124 ± 12 *
DBP (mmHg)		65 ± 8	76 ± 9.5 *
Blood Tests			
WBC (tys./μL)		7.5 ± 0.9	6.8 ± 1.7
RBC (tys./μL)		4.3 ± 0.5	4.3 ± 0.2
Hgb (g/dL)		14 ± 0.9	14 ± 1.3
Hct (%)		43 ± 2.6	42 ± 3.7
PLT (tys./μL)		250 ± 65	274 ± 60
sCre (mg/dL)		0.73 ± 0.5	0.73 ± 0.4
sUrea (mg/dL)		15 ± 10	25 ± 11
HDL (mg/dL)		52 ± 10	48 ± 12
LDL (mg/dL)		70 ± 25	97 ± 24 *
TC (mg/dL)		167 ± 45	177 ± 74
TG (mg/dL)		70 ± 38	122 ± 72 *
Glucose (mg/dL)		82 ± 5.5	89 ± 7.9
IL-6 (pg/mL)		1.44 ± 0.57	2.49 ± 0.58 *
eGFR (mL/min/1.73m^2^)		140 ± 29	118 ± 38
Pharmacotherapy			
Hypotensive drugs	0 per day *n* (%)	0 (0)	22 (39)
	1 per day *n* (%)	0 (0)	24 (45)
	2 per day *n* (%)	0 (0)	7 (13)
Angiotensin-converting enzyme inhibitors *n* (%)		0 (0)	21 (40)
Angiotensin receptor blockers *n* (%)		0 (0)	5 (9)
Calcium channel blockers *n* (%)		0 (0)	4 (7)

BMI—Body Mass Index; DBP—diastolic blood pressure; eGFR—estimated glomerular filtration rate; Hct—hematocrit; HDL—high-density lipoprotein; Hgb—hemoglobin; LDL—low-density lipoprotein; PLT—platelet; SBP—systolic blood pressure; sCre—serum creatinine; sUrea—serum Urea; TC—total cholesterol; TG—triglycerides; WBC—white blood cells. * *p* < 0.05 vs. the control group.

**Table 2:** Summary of ABPM profiles in hypertensive children.

24 h SBP (mmHg)	127 ± 11
24 h DBP (mmHg)	69 ± 7.5
SBP daytime (mmHg)	130 ± 11
DBP daytime (mmHg)	72 ± 7.6
SBP nighttime (mmHg)	117 ± 11
DBP nighttime (mmHg)	72 ± 16
SBPL daytime (%)	51 ± 3.7
DBPL daytime (%)	19 ± 3.0
SBPL nighttime (%)	47 ± 4.6
DBPL nighttime (%)	20 ± 2.8
SBP 24 h Z-score	0.21 ± 5.9
DBP 24 h Z-score	0.25 ± 2.2
SBP day Z-score	0.91 ± 2.5
DBP day Z-score	136 ± 675
SBP night Z-score	-7 ± 20
DBP night Z-score	-7.9 ± 29

ABPM—ambulatory blood pressure monitoring; DBP—diastolic blood pressure; SBP—systolic blood pressure; DBPL—diastolic blood pressure load; SBPL—systolic blood pressure load.

**Table 3:** Salivary gland function and dental examination of children with hypertension and healthy controls.

	Control *n* = 53	Hypertension *n* = 53
NWS flow (mL/min)	0.47 ± 0.05	0.43 ± 0.05
SWS flow (mL/min)	1.8 ± 0.09	0.89 ± 0.09 *
TP NWS (μg/mL)	1350 ± 185	1330 ± 264
TP SWS (μg/mL)	1019 ± 217	772 ± 228 *
α-amylase NWS (μmol/mg protein)	0.2 ± 0.08	0.19 ± 0.08
α-amylase SWS (μmol/mg protein)	0.28 ± 0.05	0.21 ± 0.08 *
DMFT	3.1 ± 0.1	3.1 ± 0.2
dmft	11 ± 0.1	11.1 ± 0.1
PBI	0 ± 0.1	0 ± 0.1
GI	0 ± 0.1	0 ± 0.1

DMFT—decay, missing, filled of permanent teeth; dmft—decay, missing, filled of milk teeth; PBI—Papilla Bleeding Index; GI—Gingival Index; NWS—non-stimulated whole saliva; SWS—stimulated whole saliva; TP—total protein content. * *p* < 0.05 vs. the control group.

**Table 4:** Correlations between salivary redox biomarkers and clinical characteristics in the control group.

Pair of Variables	r	*p*
SWS		
CAT and SOD	0.32	0.028
Saliva and blood		
AGE NWS and AGE plasma	0.83	<0.001
MDA NWS and MDA plasma	0.83	<0.001

CAT—catalase; SOD—superoxide dismutase; NWS—non-stimulated whole saliva; SWS—stimulated whole saliva.

**Table 5:** Correlations between salivary redox biomarkers and clinical characteristics in children with hypertension.

Pair of Variables	r	*p*
NWS		
SOD and TOS	0.83	<0.001
MDA and SBP	0.64	<0.001
MDA and SBP	0.49	0.001
SWS		
CAT and TOS	0.75	<0.001
UA and TOS	0.62	<0.001
UA and DBP	0.8	<0.001
AGE and SBP	0.64	<0.001
AGE and DBP	0.5	0.001
MDA and SBP	0.6	<0.001
MDA and DBP	0.31	0.025
UA and DBP	0.8	<0.001
UA and DBP 24 h Z-score	0.35	0.016
UA and SBP 24 h Z-score	0.41	0.004
GSH and Peroxynitrite	-0.61	<0.001
Peroxynitrite and MDA	0.61	<0.001
Peroxynitrite and nitrotyrosine	0.68	<0.001
SWS flow and MDA	−0.77	<0.001
SWS flow and NO	0.56	<0.001
SWS flow and peroxynitrite	−0.42	0.002
SWS flow and nitrotyrosine	−0.41	0.002
Saliva and blood		
AGE NWS and AGE plasma	0.8	<0.001
MDA NWS and MDA plasma	0.89	<0.001
UA SWS and IL-6	0.72	<0.001

AGE—advanced glycation end products; CAT—catalase; DBP—diastolic blood pressure; GSH—reduced glutathione; MDA—malondialdehyde; NO—nitric oxide; NWS—non-stimulated whole saliva; SBP—systolic blood pressure; SOD—superoxide dismutase; SWS—stimulated whole saliva; TOS—total oxidant status; UA—uric acid.

**Table 6:** Receiver operating characteristic (ROC) analysis of redox biomarkers in non-stimulated and stimulated saliva of children with hypertension and the control subjects.

	AUC	Confidence Intervals	*p* Value	Cut-off	Sensitivity (%)	Specificity (%)
NWS						
SOD (mU/mg protein)	0.91	0.86–0.96	<0.001	>1.65	77	83
CAT (nmol H_2_O_2_/min/mg protein)	0.68	0.58–0.78	0.002	>0.52	64	66
Px (mU/mg protein)	0.55	0.43–0.67	0.406	>0.49	55	64
GSH (µg/mg protein)	0.87	0.80–0.94	<0.001	<0.43	79	79
UA (µg/mg protein)	0.85	0.77–0.92	<0.001	>3.35	72	79
TAC (µmol/mg protein)	0.83	0.74–0.92	<0.001	>0.59	77	82
TOS (nmol H_2_O_2_ equiv/mg protein)	0.87	0.79–0.95	<0.001	>10.30	81	87
OSI (TOS/TAC ratio)	0.58	0.47–0.68	0.186	>13.45	57	55
AGE (AFU/mg protein)	0.96	0.92–1.00	<0.001	>2.08	92	92
MDA (µmol/mg protein)	0.79	0.70–0.87	<0.001	>101.3	75	74
NO (nmol/mg protein)	0.63	0.52–0.73	0.026	<180.6	62	58
Peroxynitrite (pmol/mg protein)	0.79	0.69–0.89	<0.001	>63.47	75	77
Nitrotyrosine (pmol/mg protein)	0.63	0.53–0.74	0.018	>221.3	64	60
SWS						
SOD (mU/mg protein)	0.74	0.65–0.84	<0.001	>3.72	74	70
CAT (nmol H_2_O_2_/min/mg protein)	0.93	0.87–0.98	<0.001	>5.11	91	91
Px (mU/mg protein)	0.79	0.70–0.88	<0.001	>0.47	77	72
GSH (µg/mg protein)	0.79	0.70–0.88	<0.001	<0.70	70	70
UA (µg/mg protein)	0.98	0.96–1.00	<0.001	>5.50	94	96
TAC (µmol/mg protein)	0.80	0.71–0.88	<0.001	>0.82	74	75
TOS (nmol H_2_O_2_ equiv/mg protein)	0.61	0.50–0.72	0.055	>19.37	55	58
OSI (TOS/TAC ratio)	0.64	0.53–0.74	0.015	<22.30	58	55
AGE (AFU/mg protein)	0.99	0.97–1.00	<0.001	>4.64	92	92
MDA (µmol/mg protein)	0.80	0.72–0.89	<0.001	>68.72	77	74
NO (nmol/mg protein)	0.83	0.75–0.91	<0.001	<197.7	74	77
Peroxynitrite (pmol/mg protein)	0.84	0.76–0.92	<0.001	>72.86	83	79
Nitrotyrosine (pmol/mg protein)	0.72	0.62–0.82	0.001	>204.5	66	66

AGE—advanced glycation end products; AUC—area under the curve; CAT—catalase; GPx—glutathione peroxidase; GSH—reduced glutathione; MDA—malondialdehyde; NWS—non-stimulated whole saliva; Px—salivary peroxidase; SOD—superoxide dismutase; SWS—stimulated whole saliva; TAC—total antioxidant capacity; TOS—total oxidant status; UA—uric acid.

**Table 7:** Receiver operating characteristic (ROC) analysis of redox biomarkers in erythrocytes/plasma of children with hypertension and the control subjects.

	AUC	Confidence Intervals	*p* Value	Cut-off	Sensitivity (%)	Specificity (%)
Erythrocytes						
SOD (mU/mg protein)	0.89	0.83–0.96	<0.001	>0.37	83	83
CAT (nmol H_2_O_2_/min/mg protein)	0.85	0.77–0.92	<0.001	>0.43	79	79
GPx (mU/mg protein)	1.00	1.00–1.00	<0.001	>0.23	100	98
Plasma						
GSH (µg/mg protein)	0.70	0.59–0.80	0.001	<3.19	64	68
UA (µg/mg protein)	0.68	0.58–0.78	0.002	>0.81	64	66
TAC (µmol/mg protein)	0.85	0.77–0.94	<0.001	>0.62	81	85
TOS (nmol H_2_O_2_ equiv/mg protein)	0.76	0.67–0.86	<0.001	>7.95	77	74
OSI (TOS/TAS ratio)	0.57	0.46–0.68	0.226	>13.45	57	55
AGE (AFU/mg protein)	1.00	1.00–1.00	<0.001	>3.96	98	100
MDA (µmol/mg protein)	0.90	0.84–0.97	<0.001	>103.7	85	92
NO (nmol/mg protein)	0.82	0.73–0.91	<0.001	<16.66	77	79
Peroxynitrite (pmol/mg protein)	0.78	0.68–0.87	<0.001	>86.27	72	74
Nitrotyrosine (pmol/mg protein)	0.67	0.57–0.77	0.003	>166.2	68	64

AGE—advanced glycation end products; AUC—area under the curve; CAT—catalase; GPx—glutathione peroxidase; GSH—reduced glutathione; MDA—malondialdehyde; NWS—non-stimulated whole saliva; Px—salivary peroxidase; SOD—superoxide dismutase; SWS—stimulated whole saliva; TAC—total antioxidant capacity; TOS—total oxidant status; UA—uric acid.
